# Safer Attitude to Risky Decision-Making in Premanifest Huntington’s Disease Subjects

**DOI:** 10.3389/fpsyg.2019.00846

**Published:** 2019-04-16

**Authors:** Giulia D’Aurizio, Simone Migliore, Giuseppe Curcio, Ferdinando Squitieri

**Affiliations:** ^1^Department of Biotechnological and Applied Clinical Sciences, University of L’Aquila, L’Aquila, Italy; ^2^Huntington and Rare Diseases Unit, Fondazione IRCCS Casa Sollievo della Sofferenza Hospital, San Giovanni Rotondo, Italy

**Keywords:** executive functions, decision making, feedback processing, dorsolateral prefrontal cortex, game of dice task, movement disorder

## Abstract

Huntington’s disease (HD) is an inherited neurodegenerative disorder characterized by involuntary, jerky movements, incoordination, behavioral changes and subtle executive and cognitive impairment starting before motor symptoms. Our study aimed to assess the risky decision-making process in premanifest (pre) HD subjects, by means Game of Dice Task (GDT). As dependent variables, several GDT outcomes have been taken into consideration. We recruited 30 subjects (15 females) with preHD (i.e., Diagnosis Confidence Level < 4; Total Motor Score < 10), and 21 age, gender and education matched neurologically normal subjects (11 females). GDT is a computer-guided task where subjects are invited to watch the digits on which to bet and to evaluate the related potential risk to win or loss. Our results showed that decision and feedback times were longer in preHD than in neurologically normal group in both disadvantageous and advantageous choices. PreHD subjects provided a greater number of “safe” strategies, taken with longer decision-making time than neurologically normal subjects, showing a reduced propensity to risk. Such behavior, characterized by increased slowness in acting and providing answers, might contribute to delineate a behavioral and cognitive profile in preHD.

## Introduction

Huntington’s disease (HD) is a rare, neurogenetic disorder caused by a CAG expanded mutation causing involuntary movements, incoordination, behavioral changes and cognitive decline. Cognitive abnormal functions may anticipate motor symptoms and may contribute to behavioral alterations (Stout et al., 2011; Bates et al., 2015).

Functional alterations depend on neurodysfunctional processes affecting striatal and cortical neurons, e.g., neocortex, hippocampus, and thalamus (Fennema-Notestine et al., 2004; Ciarmiello et al., 2006; Wolf and Klöppel, 2013) with frontal-subcortical brain circuit abnormalities (Rosas et al., 2002; Thieben et al., 2002; Kassubek et al., 2004; Douaud et al., 2006) contributing to affect executive functions and decision-making since early stages of HD.

Decision-making is a typical executive function. The Game of Dice Task (GDT) is a commonly used paradigm to examine the economic decision-making process under objective risk in a gambling situation, when explicit rules are used and information about risks and consequences of decision-making process is known (Brand et al., 2005; or a review see Liebherr et al., 2017). Cognitive dysfunction affects the decision-making process when it is under risk (Schiebener et al., 2014), as documented in other neurodegenerative diseases such as in Parkinson’s disease (PD; Brand et al., 2004; Delazer et al., 2009). To our knowledge, only one study explored GDT abilities in HD (Adjeroud et al., 2017). In this study, premanifest (pre) HD subjects performed as efficiently as control subjects, thus showing no decision-making impairment under objective risk, while manifest HD patients showed a wider executive impairment involving several abilities.

Previous studies demonstrated that performance on GDT may recruit various prefrontal cortex areas (PFC), especially dorsolateral prefrontal cortex (DLPFC; Labudda et al., 2008; Schiebener and Brand, 2015), as well as anterior cingulate cortex (ACC; Yeung and Sanfey, 2004). These brain areas showed a reduced activation in premanifest stage of HD (Domínguez et al., 2017), and these neural activation pattern could explain potentially alteration of economic decision-making process.

In our study, we aimed to assess the economic decision-making ability in preHD subjects with no neurological and cognitive impairment, in order to identify early potential differences in the decision-making process of these subjects with respect to the control group, differences that could reflect the alterations of the fronto-subcortical circuits of preHD subjects. Specifically, we expected a slowing performance in preHD group and an increased tendency toward risky decisions. To this aim, we have used the GDT to assay the economic decision-making, a paradigm which reveals executive function and feedback processing, i.e., a fundamental component of an individual daily life and of global cognitive function.

## Materials and Methods

### Participants

We recruited 30 subjects with preHD (i.e., Total Motor Score (TMS) < 10, Diagnostic Confidence Level (DCL) < 4; 15 female) (Huntington Study Group, 1996) and 21 age, gender and education matched control subjects (11 female) from January to May 2018. All subjects mutation carriers (CAG expansion ≥ 40) had performed a predictive testing and had a disease burden (i.e., CAG/Age Product score) below 400 (Penney et al., 1997; Tabrizi et al., 2009; Fusilli et al., 2018). The preHD group was assessed by the Unified Huntington’s Disease Rating Scale (UHDRS) (Huntington Study Group, 1996). Demographic and clinical characteristics are reported in Table 1. All preHD subjects were not under current treatment when included in the study and at the moment of the assessment. We excluded subjects with neurological conditions other than HD, medical condition that might influence cognition, a history of a developmental disorder [e.g., attention-deficit hyperactivity disorder (ADHD), learning disability], a history of substance or alcohol dependence or current abuse, and current or previous psychotic disorder. The participants were recruited in Rome (LIRH Foundation at CSS-Mendel Institute) and in San Giovanni Rotondo (IRCCS Casa Sollievo della Sofferenza Research Hospital). The study was designed in accordance with the ethical principles of the Declaration of Helsinki and was approved by the Institutional Review Board of the Institute “Leonarda Vaccari” of Rome (Italy). Moreover, all participants signed an informed consent before participation.

**Table 1:** Demographic and clinical characteristics (mean ± standard deviation) of the study sample.

	Premanifest HD (preHD)	Control subjects	*p*
Number	30	21	n.s.
Gender (F–M)	15 F – 15 M	11 F – 10 M	n.s.
Age (years ± SD) (range)	34,32 9,38	33,67 13,75	n.s.
	(24–55)	(22–60)	
Education	13,37 3,86	14,19 1,8	n.s.
CAG Expansion (range)	42,97 2,08		
	(40–47)		
Disease Burden Score	249,64 73,46		
TMS	5 2		
TFC	13		
FA	25		
IS	100		


*HD: Huntington Disease; TMS: Total Motor Score; TFC: Total Functional Capacity; FA: Functional Assessment; IS: Independent Scale; Disease burden score: age ^∗^ (CAG length – 35.5); n.s: not significant.*

### Game of Dice Task Assessment

Participants were tested individually in a well-lit, sound-proof room. They were seated at 50 cm from 15-inch computer monitor and asked to place 18 bets throw to a virtual die, with the aim to maximize a starting capital (1000 €). During the test, the subjects see on the screen the digits on which to bet and the related potential win or loss: a single digit associated with the probability of losing/ winning 1000 € or a combination of two, three or four numbers, respectively associated with the probability of winning/losing 500, 200 or 100 €. After each throw, the gain (congruence between the selected number or numbers and the thrown number) or the loss (in case of incongruence between the selected number or numbers and the thrown number), the capital, and the number of remaining throws are continuously displayed. To bet on a single number or the combination of two numbers is considered a disadvantageous choice (or risky), because the odds of winning are 1:6 and 2:6, respectively, while betting on the combination of three or four numbers is considered an advantageous choice (or safe), because the odds of winning are 3:6 and 4:6, respectively (Brand et al., 2004, 2006).

### Statistical Analysis

Statistical comparisons between preHD and control subjects group were carried out by means of the Chi-Square test regarding gender, and Student’s *t*-test regarding age and education (see Table 1).

Moreover, as dependent variables of the GDT, the following were considered: (1) number of disadvantageous or risky choices, (2) number of advantageous or safe choices, (3) disadvantageous choices decision and feedback time, and advantageous choices decision and feedback time, (4) total score. Different scores of GDT were compared in the two different groups (preHD vs. control subjects group) as dependent variables by one-way ANOVA. Due to multiple comparisons, Bonferroni correction was applied and the level of statistical significance was set at *p* < 0.0125.

## Results

### Game of Dice Task

With respect to number of advantageous choices, statistics revealed a significant group effect (*F*_1,49_ = 6.87; *p* = 0.01; ηp^2^= 0.12) indicating a greater number of safe strategies in the preHD group (10.30 ± 4.64) than control subjects (6.71 ± 5.04). No statistically significant differences between two groups have been observed with respect to the number of risky choices (preHD = 1.77 ± 2.14; control subjects = 2 ± 1.67).

**FIGURE 1:**
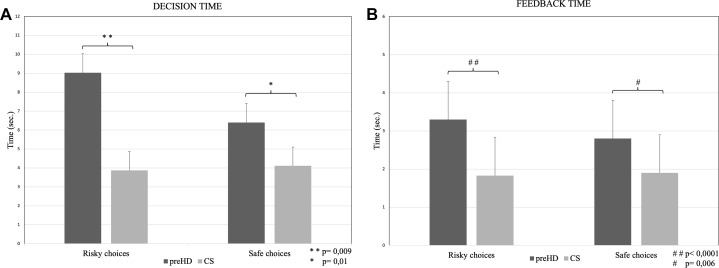
Decision time (mean ± standard deviation; panel **A**) and Feedback time (mean ± standard deviation; panel **B**) at the GDT Risky and Safe choices in premanifest HD (preHD) and control subjects (CS) groups.

With respect to decision time, statistics revealed a significant group effect for both disadvantageous (*F*_1,34_ = 7.72; *p* = 0.009; ηp^2^= 0.18, Figure 1A) and advantageous choice decision time (*F*_1,49_ = 6.58; *p* = 0.01; ηp^2^= 0.11, Figure 1A) indicating longer reaction time in preHD than in control subjects either subjects make risky choices or use safe strategy.

Statistics revealed a significant group effect in both risky feedback times (*F*_1,34_ = 15.29; *p* < 0.0001; ηp^2^= 0.31, Figure 1B) and safe feedback time (*F*_1,34_ = 8.19; *p* = 0.006; ηp^2^= 0.14, Figure 1B), indicating longer reaction times in preHD than in control subjects in both conditions.

Game of Dice Task total score did not show significant difference between the two groups.

## Discussion

Understanding the cognitive and neural basis of risky decision-making in early stage of HD could contribute to explain executive dysregulation that affect preHD subjects. The aim of our study was to assess decision-making under objective risk in preHD stage using a GDT, in an attempt to identify typical potential predictors of executive impairment that anticipate other cognitive and motor signs in HD. Regarding the expected results, we confirmed the hypothesis of a greater slowness of decision-making performance in preHD group, while, contrarily to what we hypothesized, an increase of safe decisions was observed.

Our results suggest that preHD subjects are more cautious in providing the answers to GDT than control subjects. In other terms, preHD provide safer answers than control subjects. In addition, decision time and time taken to process both disadvantageous (DT risky) and advantageous (DT safe) decisions are longer in preHD than in control subjects. Furthermore, they need more time to process feedback from the previous trial both when make a disadvantageous (FT risky) and advantageous (FT safe) decision. Such slowdown in providing the answer can be interpreted as an alteration of the ability to process the feedback from the external environment in a functional way to the selection of the next choice.

Previous studies identified a neural network involved in decision-making in the contexts of risk-taking such as GDT which includes orbitofrontal cortex (OFC), DLPFC, ventrolateral prefrontal cortex (VLPFC), ACC, insula, parietal cortex and thalamus (Ernst et al., 2002), thus lending support to the hypothesis that decision-making engages neural networks associated with different cognitive process that result altered in preHD subjects (Kirkwood et al., 2000; Johnson et al., 2007; Stout et al., 2007). More recent studies have shown that GDT performance depend on DLPFC-striatal loop activity (Krain et al., 2006) and its dysregulation affects the fronto-striatal and amygdalo-prefrontal pathways in early stage of HD (Novak and Tabrizi, 2010; Scahill et al., 2013), thus providing a possible explanation of the increase in decision time and in processing advantageous or disadvantageous feedback information. In preHD subjects, the early alteration of the prefrontal-striatal and frontal-subcortical networks (Douaud et al., 2006; Henley et al., 2008) could influence decision-making in a gambling situation; a recent study on the cognitive process involved in decision-making showed that, under objective risk conditions, performance is predicted by abilities to elaborate and categorize information, processing feedback and select an adaptive behavioral strategy (Mueller and Brand, 2018). The executive functions are beneficial to the individual behavior. Therefore, the executive dysfunction affecting preHD subjects could theoretically be prodromal to a disadvantageous behavior. Another recent electrophysiological study demonstrated that risk-taking behavior is modulated by decision context as well as by motivation (Polezzi et al., 2010).

In contrast to one previous study, our results highlight a specific executive impairment in decision-making under objective risk in a gambling situation in early stage of HD. All together these results may theoretically contribute to the executive function impairment in HD, that includes several other components such as decision-making process (Stout et al., 2001; Gleichgerrcht et al., 2010) or task-switching abilities (Migliore et al., 2018). For instance, another possible interpretation of our results suggests that HD mutation carriers may try to compensate their reaction by increasing their safe behavior to limit the consequence of their actions, thus influencing their behavior toward conservative choices.

With respect to the only one previous study that explored GDT abilities in HD (Adjeroud et al., 2017), we found some different results. These differences can be reasonably explained on the basis of some methodological dissimilarities: (1) we investigated only preHD individuals while Adjeroud and colleagues included in the study both premanifest and manifest HD (with significant differences in mean age of participants and total sample investigated); (2) clinical features of participants (TFC, TMS, disease burden, etc.) were consequently different; (3) GDT outcomes considered as index of decision making were substantially different between the two studies.

Our study has of course several limitations. As a first, our cohort is relatively limited in size: future studies will necessarily investigate risky decision-making process in greater samples even to exclude that results could be consequence of random variations. Secondarily, we selected subjects with TMS under 10. Even though they should be considered preHD individuals according to DCL < 4, disease burden score < 400 and several other, globally accepted, validated protocols (e.g., REGISTRY and ENROLL-HD platforms, worldwide observational studies for HD families), their minimal motor impairment (i.e., hand dexterity) may theoretically influence the results. We have indeed assessed the motor impairment before to perform the GDT and confirm that the hand dexterity (i.e., finger tapping and pronate/supinate-hands) mainly contributed to impair the TMS score. However, such score was never higher than 2 units per item (i.e., considering both right and left hand), that means a non-specific abnormality that might be also observed in the general population. To indirectly confirm the independence of performance by TMS we also assessed the correlation between TMS score and GDT outcomes and any significant or relevant correlation was observed. Moreover, the GDT procedure allows a comfortable subject’s index finger posture on the laptop keyboard to facilitate a prompt response; this may further reduce the possible negative effect of the clumsiness. In future investigations particular attention would be paid to the link between motor measures when assessing cognitive-behavioral outcomes. As a third potential limitation, the GDT provides a lab explanation that deserves confirmation in ecological contexts. Further studies should focus on investigating preHD subjects’ risky decision-making in an ecological context, by using a virtual or augmented reality; moreover, possible influence of behavioral aspect could be taken into account in specific future research. Finally, contrarily to the expectations we did not observe an increased tendency toward risky decisions in preHD subjects, and this could also depend by an newly developed attitude induced by the awareness of the incipient illness.

Nevertheless, our study limitations, the present findings delineate a cognitive-behavioral profile of risky decision-making in preHD subjects which may be linked to early functional alteration of the prefrontal brain cortex networks. This pattern may deserve further in depth analyses to seek potential new markers of early executive dysfunction in preHD.

## Ethics Statement

The participants were recruited in Rome (LIRH Foundation and CSS-Mendel Institute) and in San Giovanni Rotondo (IRCCS Casa Sollievo della Sofferenza Hospital), Italy. The study was designed in accordance with the ethical principles of the Declaration of Helsinki and was approved by the local ethics committee; moreover, all participants signed an informed consent before participation.

## Author Contributions

GDA and SM collected and analyzed the data. All authors designed the experiments and wrote the manuscript.

## Conflict of Interest Statement

The authors declare that the research was conducted in the absence of any commercial or financial relationships that could be construed as a potential conflict of interest.
